# Preparation of antimicrobial calcium phosphate/protamine composite powders with fluoride ions using octacalcium phosphate

**DOI:** 10.1007/s10856-022-06656-5

**Published:** 2022-04-01

**Authors:** Daisuke Koizumi, Kitaru Suzuki, Rie Togawa, Kosuke Yasui, Keishi Iohara, Michiyo Honda, Mamoru Aizawa

**Affiliations:** 1 Department of Applied Chemistry, School of Science and Technology, Meiji University, 1-1-1 Higashimita, Tama-ku, Kawasaki, Kanagawa, 214-8571 Japan; 2 Central Research Institute, Maruha Nichiro Co., 16-2 Wadai, Tsukuba, Ibaraki 300-4295 Japan; 3 Meiji University International Institute for Materials with Life Functions, 1-1-1 Higashimita, Tama-ku, Kawasaki, Kanagawa, 214-8571 Japan

## Abstract

Calcium phosphates are key biomaterials in dental treatment and bone regeneration. Biomaterials must exhibit antibacterial properties to prevent microbial infection in implantation frameworks. Previously, we developed various types of calcium phosphate powders (amorphous calcium phosphate, octacalcium phosphate (OCP), dicalcium phosphate anhydrate, and hydroxyapatite) with adsorbed protamine (which is a protein with antibacterial property) and confirmed their antibacterial property. In this study, as foundational research for the development of novel oral care materials, we synthesized calcium phosphate composite powders from three starting materials: i) OCP, which intercalates organic compounds, ii) protamine, which has antibacterial properties, and iii) F^–^ ion, which promotes the formation of apatite crystals. Through investigating the preparation concentration of the F^–^ ions and their loading into OCP, it was found that more F^–^ ion could be loaded at higher concentrations regardless of the loading method. It was also observed that the higher the preparation concentration, the more the OCP converted to fluorapatite. The synthesized calcium phosphate composite powders were evaluated for biocompatibility through proliferation of MG-63 cells, with none of the powders exhibiting any growth inhibition. Antimicrobial tests showed that the calcium phosphate composite powders synthesized with protamine and F^–^ ion by precipitation had enhanced antimicrobial properties than those synthesized by protamine adsorption. Thus, the calcium phosphate composite powder prepared from OCP, protamine, and F^–^ ion forms the basis for promising antimicrobial biomaterials.

**Figure:**
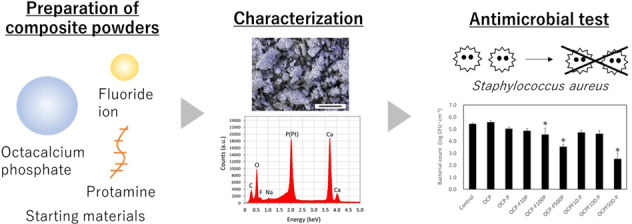
Graphical abstract

Graphical abstract

## Introduction

Calcium phosphate is an inorganic substance found abundantly in human bones and teeth, and has been extensively used as a biomaterial in dental applications and bone regeneration owing to its biocompatibility, osteoconductivity, biological stability, and mechanical strength [1]. Octacalcium phosphate (OCP), a type of calcium phosphate, is a precursor of hydroxyapatite (HAp), which is the main component of teeth and bones. However, OCP as a bone substitute exhibits distinctive properties that differ from those of HAp in terms of osteoconductivity and biodegradability [2]. The OCP is known as an interlayer compound containing a HAp layer with a dicalcium phosphate dihydrate layer, and incorporates organic compounds within its interlayer [3, 4]. Composite materials such as OCP/collagen and OCP/HAp have been reported to be safe and useful as bone substitutes in clinical studies [5, 6]. However, infection is a problem in biomaterial implantation, especially in dental implants and substitute bone; therefore, the development of antimicrobial biomaterials is underway [7, 8]. The main causative organisms of infections are the *Staphylococcus* species, including *Staphylococcus aureus* (*S. aureus*) and *Staphylococcus epidermidis* [7].

Protamine, a cationic protein that is rich in arginine residues, has a molecular weight of ~5 kDa and an isoelectric point of 12–13. Protamine is also known as salmine and clupeine, and is found bound to DNA in the sperm cells of fish, birds, and mammals. Protamine exhibits a broad spectrum of antibacterial properties against gram-positive and gram-negative bacteria and has been used as a food preservative [9–12]. Although the antimicrobial mechanisms of protamine vary from species to species and are not clearly understood, the following two mechanisms have been proposed [13]: (i) positively-charged protamine and negatively-charged extracellular epithelium act electrostatically, resulting in the release of K^+^, ATP, and intracellular enzymes, and (ii) targeting of the cell membrane and inhibition of the functions of energy transmission and nutrient uptake. In addition, protamine can regulate the transcription of bone sialoprotein, which can form HAp crystals [14] and exhibits synergistic antibacterial properties with 3-methyl-4-isopropylphenol [15].

To examine the antimicrobial properties of protamine in dental applications and bone regeneration, we previously prepared protamine-adsorbed calcium phosphate powders from various types of calcium phosphate (amorphous calcium phosphate, dicalcium phosphate anhydrate, OCP, and HAp) and confirmed their antimicrobial properties against specific microorganisms. These microorganisms included *S. aureus*, which is a type of surgery-site infection-causing bacteria, and *Streptoccus mutans*, which is a type of caries-causing bacteria [16–18]. The antimicrobial properties of protamine-adsorbed calcium phosphate powders could be ascribed to i) the action of protamine as a liquid factor released into solution and ii) the interaction of protamine adsorbed on the calcium phosphate with bacteria. In addition, the antimicrobial properties of protamine adsorbed OCP were largely attributed to protamine being immobilized on the OCP particles (Route (ii)) [18].

F^–^ ion is the ionic form of F, the thirteenth most abundant element in the earth’s crust. Approximately 99% of total F in the body is contained within bones and teeth [19]. The F^–^ ion is widely used in biomaterials as they inhibit desalination, promote remineralization and apatite crystal formation, and have antibacterial properties [20, 21]. The antimicrobial mechanism of F^–^ ion has been attributed to their influx into cells where they act on enzymatic activity and physiological processes, either directly or indirectly, inhibiting metabolism [22]. The F^–^ ion has also been reported to increase the adsorption of proteins onto OCP and HAp [23, 24].

In this study, as foundational research for the development of novel oral care materials, we focus on the preparation and characterization of calcium phosphate/protamine composite powders incorporating F^–^ ion. The powders were prepared from hydrolysis reaction of OCP (which intercalates organic compounds), with protamine (which has antibacterial properties) and F^–^ ion (which promotes the formation of apatite crystals). The resulting calcium phosphate composite powders were characterized, together with cell proliferation and antibacterial tests.

## Materials and methods

### Synthesis of OCP with and without fluoride ions

The OCP with and without F^–^ ion was prepared as previously reported [25, 26]. Briefly, 0.0335 mol of CaCO_3_, 0.1 mol of CaHPO_4_·2H_2_O, and F^–^ ion (final concentrations: 0, 10, 100, and 500 µg·cm^–3^ (as sodium fluoride: NaF)) were mixed in 1000 cm^3^ of distilled water, sonicated for 10 min, and stirred at 60 °C for 15 h. The resulting slurry was suction filtered and washed three times using distilled water. The washed slurry was then freeze dried and ground in an agate mortar to obtain the OCP powder and calcium phosphate powders containing F^–^ ion (hereafter defined as OCPF10, OCPF100, and OCPF500, where the numbers indicate the concentration of charged F^–^ ion in solution).

### Adsorption of protamine and fluoride ions on prepared calcium phosphates

Starting with 1.0 g of OCP, we added 45 cm^3^ of a 500 µg·cm^–3^ protamine solution (Protamine hydrochloride made from salmon, Maruha Nichiro Co., Japan) or 45 cm^3^ of a mixed solution of 500 µg·cm^–3^ protamine and F^–^ ion (final concentrations: 10, 100, and 500 µg·cm^–3^ (as sodium fluoride: NaF)). The resulting mixtures were stirred in a tube rotator (Fisher scientific Co. LLC, USA) for 48 h under room temperature conditions for adsorption. The precipitates were collected via centrifugation at 8600 × *g* for 10 min and washed three times using ultrapure water, and lyophilized to obtain four different powders: protamine-adsorbed calcium phosphate powder (hereafter defined as OCP-P) and calcium phosphate/protamine composite powders including F^–^ ion (hereafter defined as OCP-F10P, OCP-F100P, and OCP-F500P, where the numbers indicate the concentration of charged F^–^ ion in solution).

Emulating the above process, to 1.0 g of the calcium phosphate powder containing F^–^ ion (OCPF10, OCPF100, and OCPF500) we added 45 cm^3^ of a 500 µg·cm^–3^ protamine solution, and stirred the resulting mixtures in a tube rotator for 48 h under room temperature conditions for adsorption. The precipitates were again collected via centrifugation at 8600 × *g* for 10 min, washed three times using ultrapure water and lyophilized to obtain the calcium phosphate/protamine composite powders containing F^–^ ion (hereafter defined as OCPF10-P, OCPF100-P, and OCPF500-P, where the numbers again indicate the concentration of charged F^–^ ion in solution).

### Characterization of synthetic calcium phosphate composite powders

The crystalline phases of the synthetic calcium phosphate composite powders were identified using an X-ray diffraction (XRD) spectrometer (MiniFlex, Rigaku Co., Japan) with Cu-Kα radiation, operating at 30 kV and 15 mA. A Fourier transform infrared (FT-IR) spectrometer (NICOLET iS10, Thermo Fisher Scientific K.K., Japan) was used with the KBr method to clarify the chemical functional-groups of the resulting composite powders. The calcium/phosphorus (Ca/P) molar ratio was determined using an inductively-coupled plasma (ICP) analyzer (ICPE-9000, SHIMAZU Co., Japan). In addition, the median particle size was measured using a laser particle-size analyzer (LA-960, HORIBA Ltd., Japan). The content of fluorine in the composite powders was determined using a capillary electrophoresis instrument (PA 800 Plus, Beckman Coulter Inc., USA).

The amount of protamine adsorbed on the calcium phosphates was determined as follows: i) 0.45 cm^3^ of 1 mol·dm^–3^ HCl was added to 100 mg of the prepared calcium phosphates, and the mixtures were stirred for 1 h; ii) supernatants were collected via centrifugation at 13,000 × *g* for 1 min; and iii) protamine content in the supernatants was quantified by high-performance liquid chromatography (HPLC, Alliance, Nihon Waters K.K., Japan).

Particle morphology was assessed using scanning electron microscope (SEM) (JSM-6390LA, JEOL Ltd., Japan) at an accelerating voltage of 15 kV. The prepared powders were fixed to the stage by a piece of double-sided adhesive carbon tape and were coated with platinum. Localization of fluorine elements on the surface and elemental composition of the prepared powders were determined by energy dispersive X-ray (EDX) examination (EX-54175JMU, JEOL Ltd., Japan).

### Cell proliferation assay

Cell proliferation assay was performed according to a previously reported technique [26]. Prepared calcium phosphate composite powders were suspended within water, and the slurries were dispensed onto a 96-well tissue culture plate (353072, CORNING Inc., USA) such that each well contained 1 mg of calcium phosphate composite powder. The plates were then dried at 60 °C overnight. The dried plates were placed in a sterile bag (HM-3004, HOGY MEDICAL Co. Ltd., Japan) and sterilized using ethylene oxide gas. The MG-63 cells (IFO50108, National Institutes of Biomedical Innovation, Health and Nutrition, Japan) were seeded to each well at a cell density of 1 × 10^4^ cells·well^–1^ and cultured for 2 days at 37 °C with 5% CO_2_ in 200 µL of E-MEM medium (FUJIFILM Wako Pure Chemical Co., Japan). The used E-MEM medium contained non-essential amino acid solution (Sigma-Aldrich, Inc., USA), 10% fetal bovine serum (FBS, NICHIREI BIOSCIENCES Inc., Japan), and penicillin-streptomycin solution (FUJIFILM Wako Pure Chemical Co., Japan). Cell proliferation was evaluated by WST-8 assay (DOJINDO LABORATORIES Co., Ltd., Japan) at both 1 and 2 days after seeding (labeled Day 1 and Day 2, respectively).

### Evaluation of antimicrobial properties

Each of the calcium phosphate composite powders was placed in a sterile bag (HM-3002, HOGY MEDICAL Co. Ltd., Japan) and sterilized using ethylene oxide gas. Subsequently, 100 mm^3^ slurry of each calcium phosphate composite powder with sterile water (final concentration: 2 mg·cm^–3^) was added to test tubes with 9 cm^3^ of 9.57 mmol·dm^–3^ phosphate buffered salts (PBS, Takara Bio Inc., Japan) and 1 cm^3^ of *S. aureus* FDA209 suspended in PBS (final concentration: 10^5^ CFU·cm^–3^). Each test tube was incubated at 30 °C for 6 h with shaking at 180 rpm. The cultured medium suspensions containing *S. mutans* and the calcium phosphate composite powders were then seeded on Rapid Aerobic Count Plates (3M Japan Ltd., Japan). After incubation at 35 °C for 24 h, the colonies on the plate were counted. The cultured supernatants were filtered through a 0.22 µm pore size filter, and the protamine content in the supernatants was quantified using the Bradford method (FUJIFILM Wako Pure Chemical Corporation, Japan).

### Statistical analysis

Data were statistically evaluated using one-way analysis of variance, followed by a Tukey post-hoc test, and the results were expressed as mean ± standard deviation.

## Results and discussion

### Characterization of the prepared calcium phosphate composite powders

**Fig. 1:**
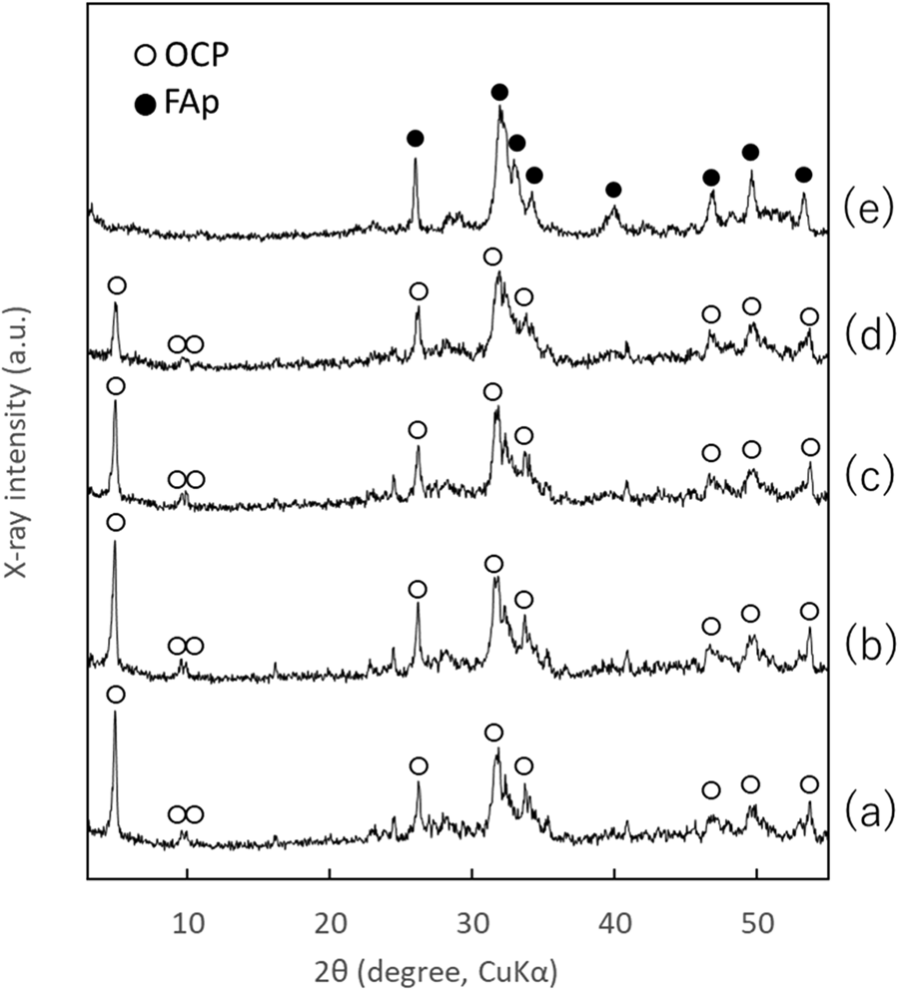
XRD patterns of calcium phosphates with and without F^–^ ion added during any protamine adsoprtion: **a** OCP, **b** OCP-P, **c** OCP-F10P, **d** OCP-F100P, and **e** OCP-F500P

**Fig. 2:**
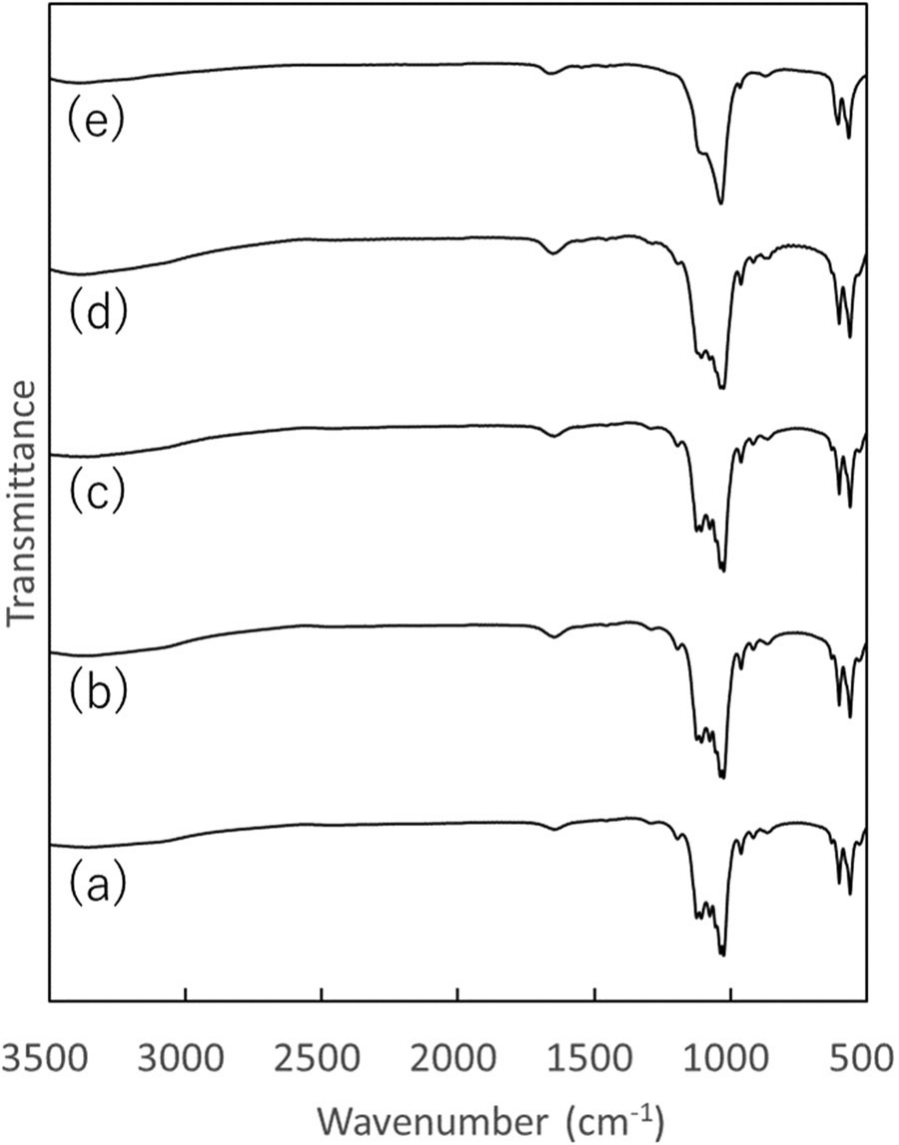
FT-IR spectra of calcium phosphates with and without F^–^ ion added during any protamine adsoprtion: **a** OCP, **b** OCP-P, **c** OCP-F10P, **d** OCP-F100P, and **e** OCP-F500P

The XRD patterns and FT-IR spectra of OCP, OCP-P, and the calcium phosphate/protamine composite powders containing F^–^ ion obtained using the adsorption method (OCP-F10P, OCP-F100P, and OCP-F500P) are shown in Figs. 1 and 2, respectively.

In the XRD patterns (Fig. 1) of OCP, OCP-P, OCP-F10P, and OCP-F100P, diffraction peaks were observed at 2*θ* = 4.9°, 9.6°, and 9.9°, which are characteristic of OCP, while OCP-F500P exhibited diffraction peaks at 2*θ* = 31.9°, 32.9°, and 39.9°, which are characteristic of fluorapatite (FAp). In the FT-IR spectra (Fig. 2), OCP, OCP-P, OCP-F10P, and OCP-F100P exhibited four P–O bands at ~1125, 1076, 1037, and 1025 cm^–1^, which are characteristic of OCP, as well as two sharp bands at 560–600 cm^–1^ corresponding to crystalline calcium phosphate of *ν*_4_ PO_4_^3–^. In contrast, OCP-F500P exhibited FAp-specific bands of *ν*_3_ PO_4_^3–^ at ~1034 cm^–1^, *ν*_1_ PO_4_^3–^ at ~962 cm^–1^, and *ν*_4_ PO_4_^3–^ at ~604 and 565 cm^–1^.

Collectively, the XRD patterns and FT-IR spectra indicate that OCP-P, OCP-F10P, and OCP- F100P maintain the crystalline phase of OCP after adsorption of F^–^ ion and protamine, whereas OCP-F500P is converted to FAp.

**Fig. 3:**
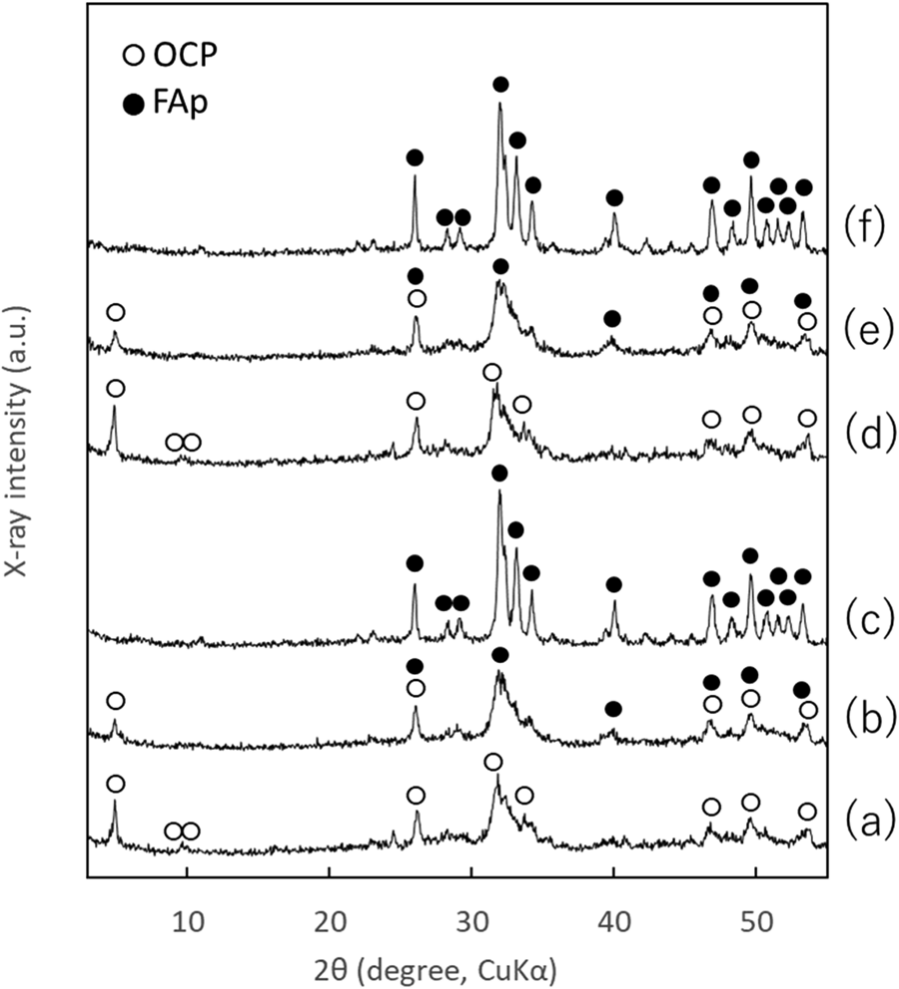
XRD patterns of calcium phosphates with F^–^ ion included in precipitation before any protamine adsorption: **a** OCPF10, **b** OCPF100, **c** OCPF500, **d** OCPF10-P, **e** OCPF100-P, and **f** OCPF500-P

**Fig. 4:**
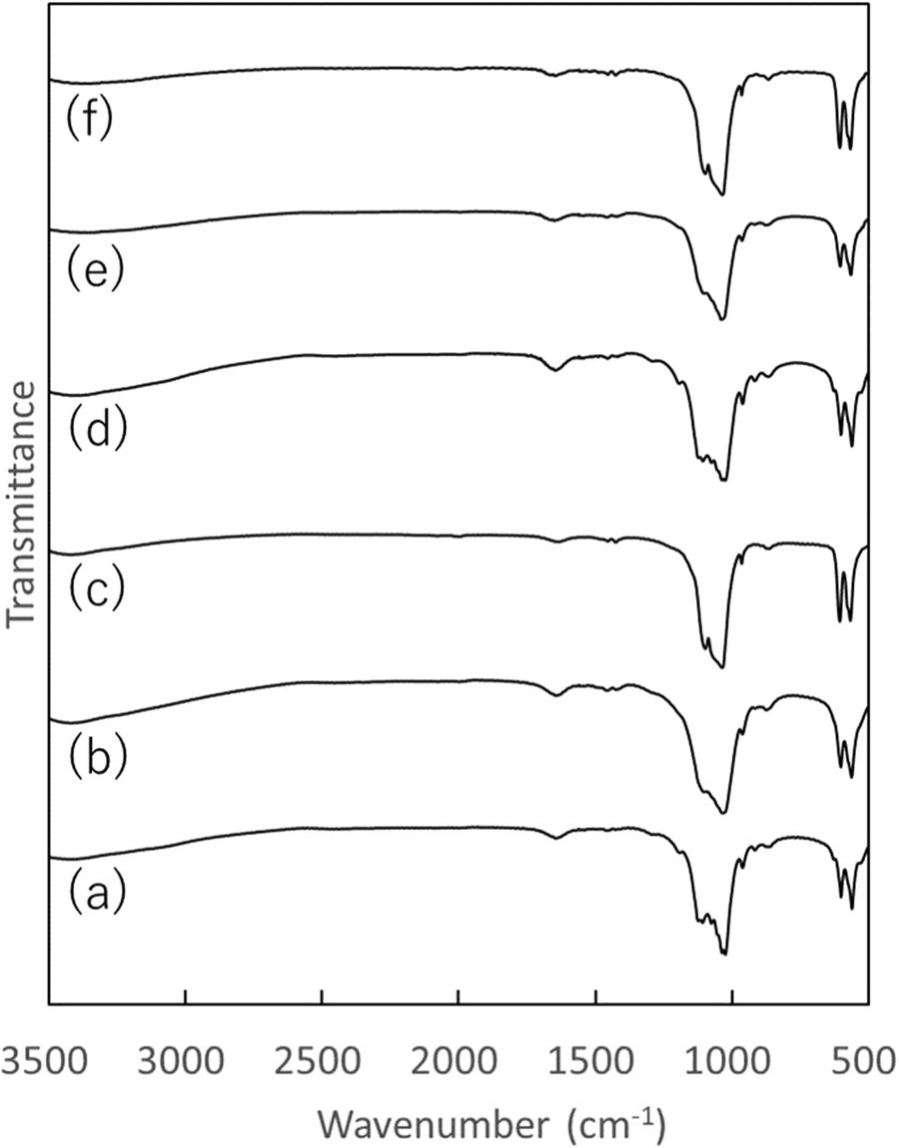
FT-IR spectra of calcium phosphates with F^–^ ion included in precipitation before any protamine adsorption: **a** OCPF10, **b** OCPF100, **c** OCPF500, **d** OCPF10-P, **e** OCPF100-P, and **f** OCPF500-P

The XRD patterns and FT-IR spectra of OCPF10, OCPF100, and OCPF500, and OCPF10-P, OCPF100-P, and OCPF500-P, to which F^–^ ion was added by the precipitation method and protamine was added by the adsorption method, are shown in Figs. 3 and 4, respectively. The XRD patterns (Fig. 3) of OCPF10 and OCPF10-P showed diffraction peaks at 2*θ* = 4.9°, 9.6°, and 9.9°, which are characteristic of OCP, while OCPF100 and OCPF100-P showed a single diffraction peak at 2*θ* = 4.9°, which is also characteristic of OCP. In contrast, the XRD patterns of OCPF500 and OCPF500-P showed diffraction peaks at 2*θ* = 31.9°, 33.1°, and 40.0°, which are characteristic of FAp.

In the FT-IR spectra (Fig. 4), OCPF10 and OCPF10-P exhibited four P–O bands at ~1125, 1076, 1037, and 1025 cm^–1^, which are characteristic of OCP, and two sharp bands at 560–600 cm^–1^ correspond to crystalline calcium phosphate of *ν*_4_ PO_4_^3–^. Contrastingly, OCPF100, OCPF100-P, OCPF500, and OCPF500-P showed FAp-specific bands of *ν*_3_ PO_4_^3–^ at ~1034 cm^–1^, *ν*_1_ PO_4_^3–^ at ~962 cm^–1^, and *ν*_4_ PO_4_^3–^ at ~604 and 565 cm^–1^.

The XRD patterns and FT-IR spectra showed that F^–^ ion was incorporated into OCP in OCPF10 and OCPF100, but in OCPF500, F^–^ ion was incorporated into FAp. Protamine adsorption appeared to cause negligible change in the crystalline phase in any of the samples, as there was little change in the XRD patterns and FT-IR spectra before and after this process.

In this study, two methods of loading F^–^ ion into OCP were assessed: one was to add F^–^ ion solutions to OCP powder and adsorb it (the adsorption method), and the other was to synthesize OCP in the presence of F^–^ ion (the precipitation method). The concentration of the charged F^–^ ion solutions (10, 100, and 500 µg·cm^–3^) were also examined. Prior study has reported that F^–^ ion promotes the conversion of OCP crystals into apatitic crystals [22]. In the two methods used in this study, when the concentration of the charged F^–^ ion solutions was at 10 and 100 µg·cm^–3^, the diffraction peak at 2*θ* = 4.9° in the XRD patterns confirmed that the crystalline phase of OCP was maintained. Conversely, when the concentration of the charged F^–^ ion solution was 500 µg·cm^–3^, the diffraction peak at 2*θ* = 4.9° was not observed in the XRD patterns, indicating that the OCP was converted to FAp. Under the present test conditions, the concentration of the charged F^–^ ion solution contributed more strongly to the conversion from OCP to FAp than the differences in the actual methods used. Although the synthesis method of OCP is different in this study compared to the method used by Shiwaku et al., the results obtained are similar [26].

The Ca/P molar ratio, median size, fluorine content, and protamine content of the prepared calcium phosphate powders are shown in Table 1. The Ca/P molar ratio of OCP was larger than the theoretical value of 1.33 [25], which was due to the partial formation of HAp during the OCP formation process [25]. The Ca/P molar ratio of the calcium phosphate powders containing F^–^ ion increased with increasing concentrations of the charged F^–^ ion solutions in both the adsorption (OCP-F10P, OCP-F100P, and OCP-F500P) and precipitation (OCPF10-P, OCPF100-P, and OCPF500-P) methods. This can be attributed to the accelerated conversion of OCP (theorical Ca/P molar ratio = 1.33) to FAp (theorical Ca/P molar ratio = 1.67) with the increase in concentration of the charged F^–^ ion solution. The XRD patterns and FT-IR spectra also indicated that the conversion of OCP to FAp was accelerated with the increase in concentration of the charged F^–^ ion solution (Figs. 1–4).

**Table 1:** Ca/P molar ratio, median size, F content, and protamine content of the prepared calcium phosphates

Calcium phosphates	Ca/P molar ratio^a^	Median size (µm)	F content (mass%)^a^	Protamine content (mg·g^–1^)^a^
OCP	1.42 ± 0.01	16.1	N.D.^b^	N.D.^b^
OCP-P	1.42 ± 0.01	17.3	N.D.^b^	5.77 ± 0.05
OCP-F10P	1.42 ± 0.01	14.9	N.D.^b^	7.41 ± 0.08
OCP-F100P	1.46 ± 0.01	18.1	0.65 ± 0.02	14.8 ± 0.14
OCP-F500P	1.58 ± 0.01	16.5	3.22 ± 0.15	24.2 ± 0.09
OCPF10-P	1.45 ± 0.00	19.7	N.D.^b^	7.26 ± 0.01
OCPF100-P	1.50 ± 0.01	17.6	0.64 ± 0.04	7.23 ± 0.01
OCPF500-P	1.63 ± 0.01	10.6	3.78 ± 0.17	8.22 ± 0.12

^a^Mean ± standard deviation (*n* = 3)

^b^Not detected

The median size of the calcium phosphate powders ranged from 10.6–19.7 µm, with no large difference, but OCPF500-P had the smallest measured particle size.

Fluorine content was only detected in OCP-F100P, OCP-F500P, OCPF100-P, and OCPF500-P, ranging from 0.64–3.78 mass% fluorine.

Protamine content ranged between 7.41–22.4 mg·g^–1^ in OCP-F10P, OCP-F100P, and OCP-F500P, whose F^–^ ion was loaded by the adsorption method, and where higher F^–^ ion content was related to higher protamine content. For OCPF10-P, OCPF100-P, and OCPF500-P, whose F^–^ ion was loaded by the precipitation method, protamine content ranged from 7.23–8.22 mg·g^–1^, and there was no correlation between F^–^ ion content and protamine content.

Qiao et al. reported that F^–^ ion increased the amount of protein adsorbed on porcine bone [23]. Shiwaku et al. evaluated the amount of cytochrome c adsorbed on OCP-derived calcium phosphates, which were loaded with F^–^ ion. They reported that when F^–^ ion was loaded into the OCP-derived calcium phosphates by the adsorption method, the amount of cytochrome c adsorbed on OCP-derived calcium phosphates increased. However, when F^–^ ion was loaded into OCP-derived calcium phosphates by the precipitation method, the amount of cytochrome c adsorbed on OCP-derived calcium phosphates did not increase [24]. The reason for the increase in adsorbed protein when F^–^ ion was loaded via adsorption rather than precipitation was not clarified. However, in this study, it was considered that this may be attributed to the difference in localization of F^–^ ion between the adsorption and precipitation methods. In other words, F^–^ ion loaded via adsorption were mainly localized on the surface of the calcium phosphate crystals, while F^–^ ion loaded via precipitation were localized on both the surface and the inside of the calcium phosphate crystals. Overall, differences in the binding modes of the F^–^ ion, protamine, and calcium phosphate affected the amount of protamine adsorption.

**Fig. 5:**
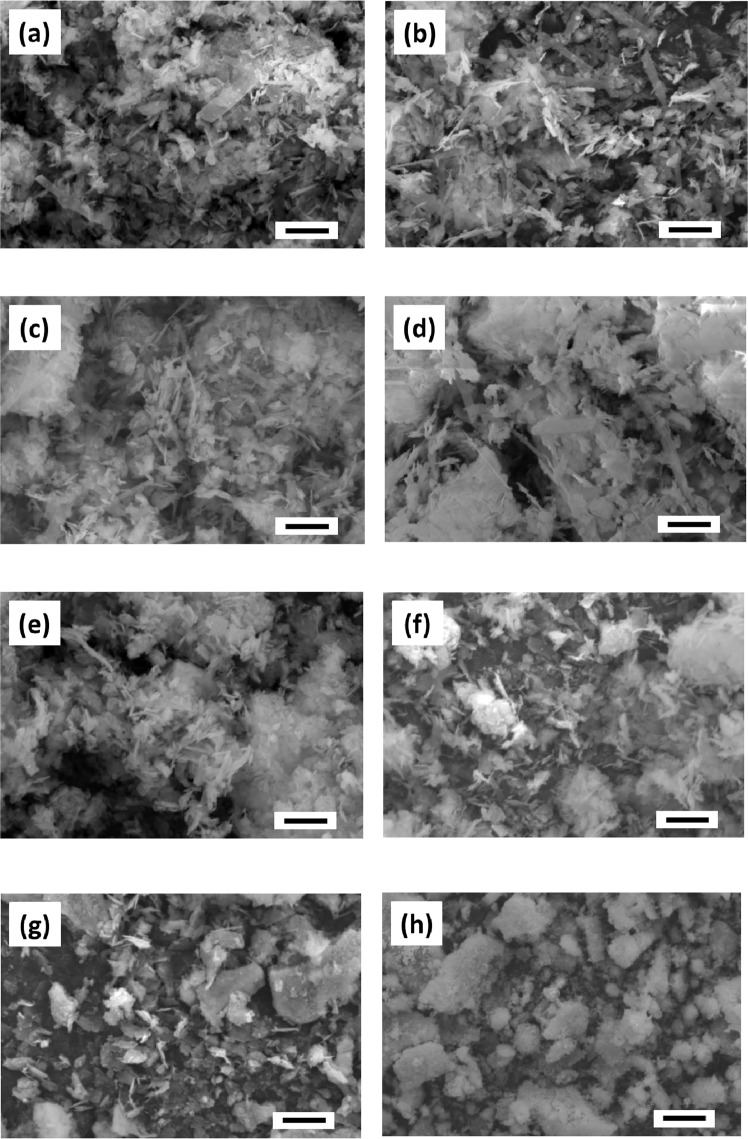
SEM micrographs of the prepared calcium phosphates: **a** OCP, **b** OCP-P, **c** OCP-F10P, **d** OCP-F100P, **e** OCP-F500P, **f** OCPF10-P, **g** OCPF100-P, and **h** OCPF500-P (Scale bars indicate 5 μm)

SEM images of the prepared calcium phosphate powders are shown in Fig. 5. Plate- and needle-like crystals were observed in both OCP and OCP-P (Fig. 5a, b, respectively). In the SEM images of calcium phosphate powders, whose F^–^ was added by the adsorption method (Fig. 5c–e), the particles became finer, and the degree of agglomeration increased as the F^–^ ion concentration increased; for OCP-F500P (Fig. 5e), the plate-like crystals observed in OCP were hardly evident. In the SEM images of calcium phosphate powders, whose F^–^ ion was added by the precipitation method (Fig. 5f–h), the particles of OCPF10-P and OCPF100-P became finer as the F^–^ ion concentration increased, and the smallest particles of OCPF500-P could hardly be observed. In the SEM images of OCPF100-P and OCPF500-P (Fig. 5g, h, respectively), a precipitation-like phenomenon was observed on the surface of large aggregates. Compared to OCP, OCPF500-P exhibited the largest change in morphology, which was attributed to the highest crystallinity, as interpreted from both the XRD patterns and IR spectra.

**Fig. 6:**
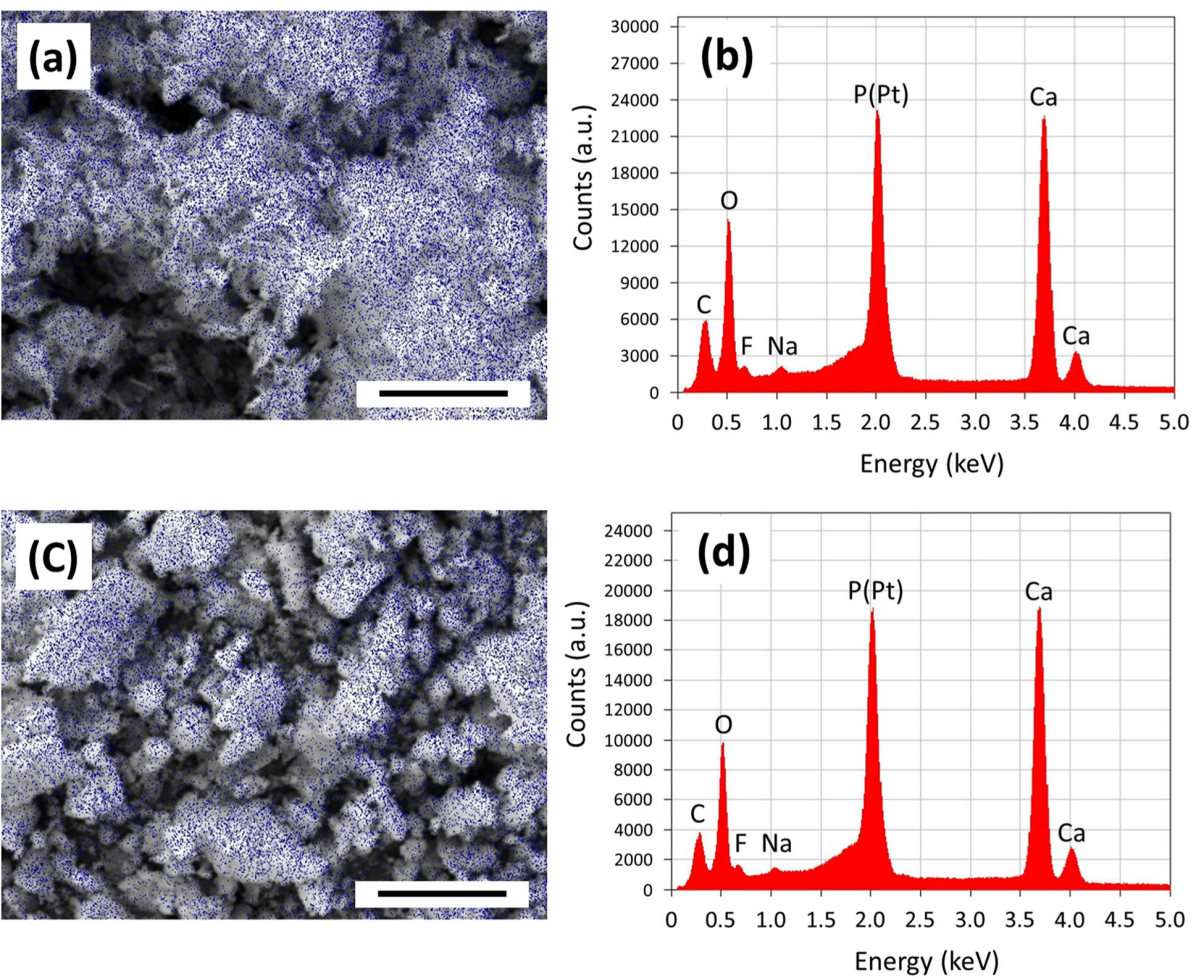
Mappings images of the F element and matching EDX spectra for selected calcium phosphate composite powders: **a**, **b** OCP-F500P and **c**, **d** OCPF500-P (Scale bars indicate 10 μm)

The mapping images of fluorine elements and matching EDX spectra are shown in Fig. 6 for OCP-F500P (Fig. 6a, b, respectively) and OCPF500-P (Fig. 6c, d, respectively). These samples were chosen for distinction as they had the highest F^–^ ion content among the F^–^ ion added by the adsorption and precipitation methods. The images (Fig. 6a, c) show that the F^–^ ion is homogeneously distributed on the surface of the particles for both calcium phosphate powders. The EDX spectra (Fig. 6b, d) show that both powders have elemental compositions of C, O, F, S, P, and Ca. The C-peak detected may be attributed to carbon tape and protamine in origin. The Na-peak detected was considered to be derived from the NaF used as the source of F^–^ ion. The P-peak indicated that the prepared powders involve P element, and there also appears to be a probable Pt-peak denoted “P (Pt)”.

### Cell proliferation assay

**Fig. 7:**
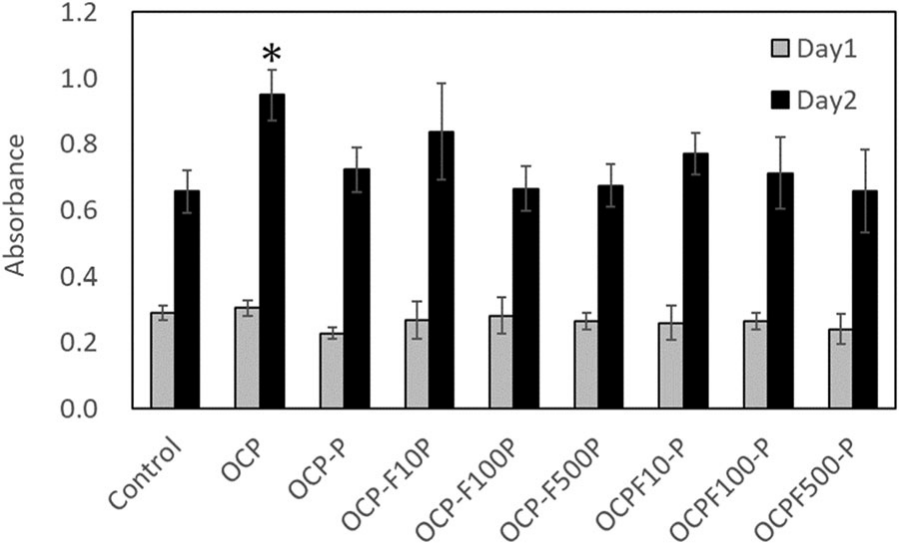
Proliferation assay of the prepared calcium powders (*n* = 4). (*p < 0.05 compared with control on the same day)

The results of the cell proliferation assay are shown in Fig. 7. Compared to the control without calcium phosphate coating on the well, the powders show comparable growth of MG-63 cells at Day 1 and Day 2 after cell seeding, and no inhibition of cell growth. Although the cell proliferation of calcium phosphates containing protamine and F^–^ ion was significantly lower than that of OCP, it was comparable to that of the control, indicating the advantage of incorporating antibacterial properties in calcium phosphate.

### Evaluation of antimicrobial property

**Fig. 8:**
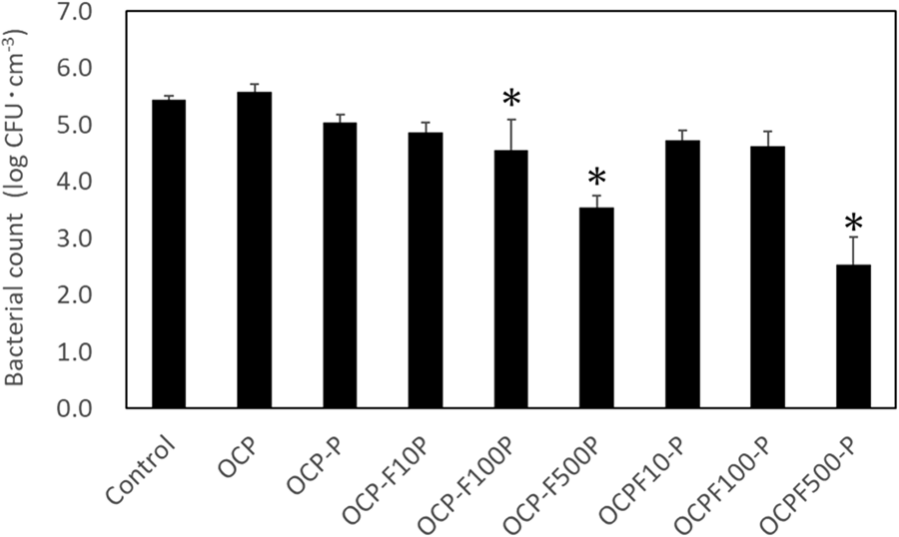
Antimicrobial properties of prepared calcium phosphates for *Staphylococcus aureus* (*S. aureus*) (*n* = 3) (*p < 0.05 compared with control)

The results of the antimicrobial test are shown in Fig. 8, where the vertical axis is in the standard logarithm, for which a decrease in value from 5 to 4 represents a decrease from 100,000 to 10,000 bacterial counts. Although the OCP did not show any decrease in the number of bacteria in comparison to the control, powders containing protamine and F^–^ ion did, especially OCPF500-P, which exhibited the best antimicrobial properties. Compared to OCP-P containing only protamine, calcium phosphate powders containing both protamine and F^–^ ion showed a greater decrease in the number of bacteria. Based on the reported antibacterial properties of F^–^ ion [19, 22], it is considered that the addition of F^–^ ion in the calcium-phosphate composite powders not only increased crystallinity, but also enhanced the antibacterial properties.

**Fig. 9:**
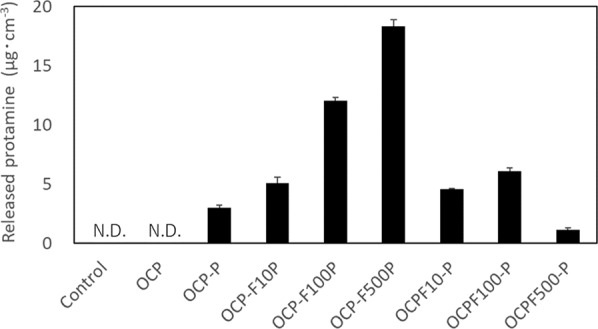
Concentration of protamine released in the cultured supernatants after incubation of *S. aureus* for 6 h (*n* = 3)

The concentration of protamine in the cultured supernatant is shown in Fig. 9. The concentration of protamine released in the cultured supernatants of calcium phosphates containing protamine ranged from 1.1–18.4 µg·cm^–3^. The OCP-F100P and OCP-F500P, which contained the highest amount of protamine, also released higher amounts of protamine than other calcium phosphate powders. Interestingly, OCPF500-P, which contained higher amount of protamine than OCP-P, OCP-F10P, OCPF10-P, and OCPF100-P, released the lowest amount of protamine overall.

The antimicrobial properties of calcium phosphate composite powders can be attributed to both the action of protamine and F^–^ ion as a liquid factor released into solution, and interaction of protamine and F^–^ ion adsorbed on calcium phosphate with bacteria. Although the reasons for the highest antimicrobial properties being recorded for OCPF500-P were not clear, it is considered that the addition of F^–^ ion changed the crystallinity most significantly in this case owing to the differences in the binding modes and adsorption/release properties of protamine and the F^–^ ion. In summary, the calcium phosphate composite powders prepared using OCP, protamine, and F^–^ ion as precipitate materials exhibited enhanced antimicrobial activity than that of protamine-adsorbed OCP, (especially OCPF500-P, which exhibited the best antimicrobial properties), and are thus considered as promising antimicrobial materials.

## Conclusions

As foundational research for the development of novel oral care materials, we focused on three materials: i) OCP, which intercalates organic compounds, ii) protamine, which has antibacterial properties, and iii) F^–^ ion, which promote the formation of apatite crystals. Calcium-phosphate composite powders were prepared from these materials by precipitation and adsorption methods, and their morphology, cell proliferation, and antimicrobial properties were investigated. It was confirmed that higher concentrations of F^–^ ion led to more ions being loaded into OCP, and more OCP was converted to fluorapatite. Cell proliferation tests showed no inhibition of MG-63 cells. Antimicrobial tests showed that calcium phosphate powders containing protamine and F^–^ ion prepared by precipitation had enhanced antimicrobial properties than those of protamine-adsorbed OCP, and are thus considered as promising antimicrobial materials.

## References

[1] Jeong J, Kim JH, Shim JH, Hwang NS, Heo CY (2019). Bioactive calcium phosphate materials and applications in bone regeneration. Biomater Res..

[2] Suzuki O, Shiwaku Y, Hamai R (2020). Octacalcium phosphate bone substitute materials: comparison between properties of biomaterials and other calcium phosphate materials. Dent Mater J..

[3] Monma H, Nishimura Y, Okura T (2005). Characterization of layer-structured octacalcium phosphate/dicarboxylate composite. Phosphorus Res Bull..

[4] Yokoi T, Goto T, Hara M, Sekino T, Seki T, Kamitakahara M (2021). Incorporation of tetracarboxylate ions into octacalcium phosphate for the development of next-generation biofriendly materials. Commun Chem..

[5] Kim JS, Jang TS, Kim SY, Lee WP (2021). Octacalcium phosphate bone substitute (Bontree^®^): from basic research to clinical case study. Appl Sci..

[6] Kawai T, Shinji Kamakura S, Matsui K, Fukuda M, Takano H, Iino M (2020). Clinical study of octacalcium phosphate and collagen composite in oral and maxillofacial surgery. J Tissue Eng.

[7] Wekwejt M, Dziaduszewska M, Pałubicka A. The problem of infections associated with implants—an overview. Eur J Med Technol 2018;4:19–26.

[8] Francolini I, Vuotto C, Piozzi A, Donelli G (2017). Antifouling and antimicrobial biomaterials: an overview. APMIS.

[9] Gill TA, Singer DS, Thompson JW (2006). Purification and analysis of protamine. Process Biochem.

[10] Islam NM, Itakura T, Motohiro T (1984). Antibacterial spectra and minimum inhibition concentration of clupeine and salmine. Bull Jpn Soc Sci Fish..

[11] Suzuki K, Ando T (1972). Studies on protamines: XVII. The complete amino acid sequence of clupeine YI. J Biochem.

[12] Miller BF, Abrams R, Dorfman A, Klein M (1942). Antibacterial properties of protamine and histone. Science.

[13] Kim YH, Kim SM, Lee SY (2015). Antimicrobial activity of protamine against oral microorganisms. Biocontrol Sci.

[14] Zhou L, Matsumura H, Mezawa M, Takai H, Nakayama Y, Mitarai M, Ogata Y (2013). Protamine stimulates bone sialoprotein gene expression. Gene.

[15] Fujiki M, Honda M (2020). The investigation of synergistic activity of protamine with conventional antimicrobial agents against oral bacteria. Biochem Biophys Res Commun..

[16] Fujiki M, Abe K, Hayakawa T, Yamamoto T, Torii M, Togawa R (2019). Antimicrobial activity of protamine-loaded calcium phosphates against oral bacteria. Materials.

[17] Honda M, Matsumoto M, Aizawa M (2020). Potential application of protamine for antimicrobial biomaterials in bone tissue engineering. Int J Mol Sci.

[18] Koizumi D, Suzuki K, Minamisawa H, Togawa R, Yasui K, Iohara K, Honda M, Aizawa M. Preparation of protamine-adsorbed calcium phosphate powders and their antibacterial property. J Asian Ceram Soc. 2022;10:230–40. 10.1080/21870764.2022.2035488

[19] Antoine A, Farah D, Sibelle AH, Jacqueline D (2018). The fluoride debate: the pros and cons of fluoridation. Prev Nutr Food Sci.

[20] Neti B, Sayana G, Muddala L, Mantena SR, Yarram A, GVD H (2020). Fluoride releasing restorative materials: a review. Int J Dent Mater..

[21] Aoba T (1997). The effect of fluoride on apatite structure and growth. Crit Rev Oral Biol Med..

[22] Liao Y, Brandt BW, Li J, Crielaard W, Loveren CV, Deng DM (2017). Fluoride resistance in *Streptococcus mutans*: a mini review. J Oral Microbiol..

[23] Qiao W, Liu Q, Li Z, Zhang H, Chen Z (2017). Changes in physicochemical and biological properties of porcine bone derived hydroxyapatite induced by the incorporation of fluoride. Sci Technol Adv Mater..

[24] Shiwaku Y, Honda Y, Anada T, Morimoto S, Masuda T, Sasaki K, Suzuki O (2010). Analysis of physicochemical properties of octacalcium phosphate prepared by hydrolysis and co-precipitation with fluoride ions. J Ceram Soc Japan..

[25] Ban S, Hasegawa J, Maruno S (1996). Synthesis of octacalcium phosphate and its transformation to apatite. JSDMD.

[26] Shiwaku Y, Anada T, Yamazaki H, Honda Y, Morimoto S, Sasaki K, Suzuki O (2012). Structural, morphological and surface characteristics of two types of octacalcium phosphate-derived fluoride-containing apatitic calcium phosphates. Acta Biomater..

